# From receptor binding kinetics to signal transduction; a missing link in predicting *in vivo* drug-action

**DOI:** 10.1038/s41598-017-14257-4

**Published:** 2017-10-26

**Authors:** Indira Nederpelt, Maria Kuzikov, Wilbert E. A. de Witte, Patrick Schnider, Bruno Tuijt, Sheraz Gul, Adriaan P. IJzerman, Elizabeth C. M. de Lange, Laura H. Heitman

**Affiliations:** 1 Division of Medicinal Chemistry, Leiden Academic Centre for Drug Research (LACDR), Leiden University, P.O. Box 9502, 2300 RA Leiden, The Netherlands; 2 Fraunhofer IME Screening Port, Schnackenburgallee 114, D-22525 Hamburg, Germany; 3 Roche Pharmaceutical Research and Early Development, Small Molecule Research, Roche Innovation Center Basel, F. Hoffmann-La Roche Ltd, Grenzacherstrasse 124, 4070 Basel, Switzerland; 4 Division of Pharmacology, Leiden Academic Centre for Drug Research (LACDR), Leiden University, P.O. Box 9502, 2300 RA Leiden, The Netherlands

## Abstract

An important question in drug discovery is how to overcome the significant challenge of high drug attrition rates due to lack of efficacy and safety. A missing link in the understanding of determinants for drug efficacy is the relation between drug-target binding kinetics and signal transduction, particularly in the physiological context of (multiple) endogenous ligands. We hypothesized that the kinetic binding parameters of both drug and endogenous ligand play a crucial role in determining cellular responses, using the NK1 receptor as a model system. We demonstrated that the binding kinetics of both antagonists (DFA and aprepitant) and endogenous agonists (NKA and SP) have significantly different effects on signal transduction profiles, i.e. potency values, *in vitro* efficacy values and onset rate of signal transduction. The antagonistic effects were most efficacious with slowly dissociating aprepitant and slowly associating NKA while the combination of rapidly dissociating DFA and rapidly associating SP had less significant effects on the signal transduction profiles. These results were consistent throughout different kinetic assays and cellular backgrounds. We conclude that knowledge of the relationship between *in vitro* drug-target binding kinetics and cellular responses is important to ultimately improve the understanding of drug efficacy *in vivo*.

## Introduction

**Figure 1:**
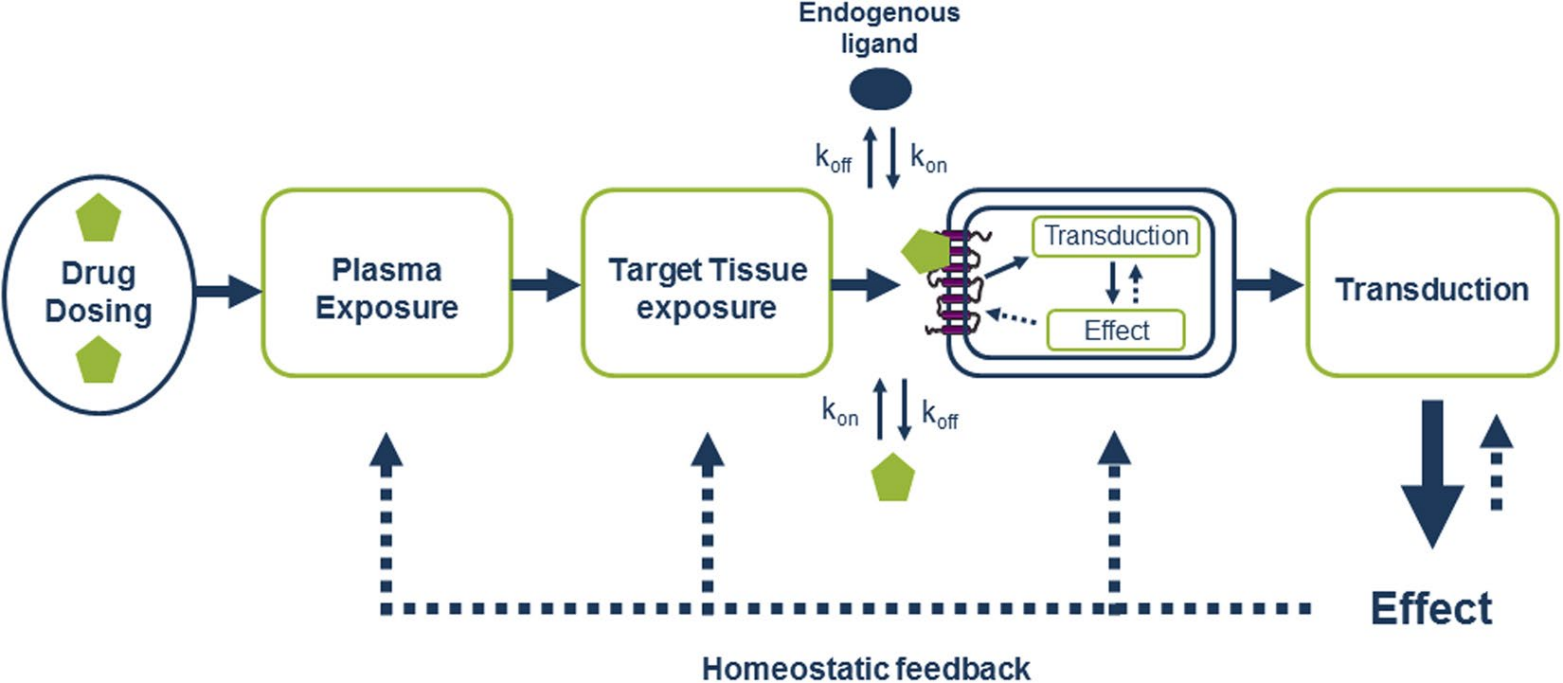
Schematic overview of factors involved between drug dosing and body responses, i.e. drug effects. While drug dosing, plasma pharmacokinetics, target tissue distribution, intra-tissue or target site distribution, cellular signal transduction and body responses are often examined drug-target binding kinetics are often disregarded. More importantly, elucidation of the pivotal step, i.e. effects of binding kinetics on signal transduction, from drug-target binding kinetics to *in vivo* drug responses is highly desirable.

Drug discovery is consistently challenged with overcoming high attrition rates due to lack of efficacy and safety in clinical trials. In the past decade, numerous researchers have proposed drug-target binding kinetics (i.e. association and dissociation rates) as important *in vitro* parameters and have suggested to include these early in the drug discovery paradigm^1–4^. While plasma pharmacokinetic profiles are relatively well understood, and progress is made in understanding and predicting target tissue distribution and target occupancy^5–7^, the crucial step from drug-target binding kinetics to the *in vivo* cellular response that precedes the entire body’s response is typically missing (Fig. 1). Since these responses cannot yet be measured in the living body, we resort to using *in vitro* systems that reflect the *in vivo* conditions as closely as possible. So far, numerous receptor binding assays, such as radioligand binding^8^, surface plasmon resonance (SPR), surface acoustic wave (SAW)^9^, and time-resolved fluorescence resonance energy transfer (TR-FRET) assays^10^, have been designed and validated to study binding kinetics at the receptor level. However, there is a need for kinetic functional assays to better predict *in vivo* cellular responses of kinetically diverse compounds. Functional assays that are well suited for this purpose include the real-time GloSensor™ cAMP assay^11,12^, measuring cAMP production, and the real-time impedance-based xCELLigence™ assay^13,14^, that measures changes in cell morphology as a more integral cellular response.

The neurokinin 1 (NK1) receptor is an example of a target for which drugs with optimal binding kinetics are reported. It is mainly expressed in the central nervous system (CNS) and has been suggested to play a role in the regulation of affective behavior and emesis in the brain, as well as nociception in the spinal cord^15,16^. While a plethora of NK1 antagonists have been synthesized^17–19^, most antagonists have failed in the clinic due to a reported lack of efficacy^20,21^. Currently, two small molecule NK1 antagonists are marketed to treat chemotherapy-induced emesis and nausea, namely aprepitant and netupitant. A study by Hale *et al*. indicated that aprepitant is superior to other NK1 receptor antagonists due to its slow receptor dissociation rate of 0.0054 min^−1^ at room temperature^22^. These results were confirmed in a later study in which the long-lasting *in vivo* effects of aprepitant were directly related to its slow dissociation rate rather than a long half-life^23^. More recently, the highly selective NK1 antagonist netupitant, in combination with a serotonin 5-HT_3_ receptor antagonist, was approved by the FDA^24^. Similar to aprepitant, netupitant was considered insurmountable, i.e. able to depress the maximal agonist-induced response by preventing agonist rebinding, and shown resistant to wash-out experiments, i.e. during wash-out netupitant was still tightly bound to the receptor^25^. The authors proposed slow receptor dissociation kinetics as the mechanism hereof.

Another important aspect in *in vivo* receptor binding is competition of the drug with endogenous ligands. It is therefore crucial to study the binding kinetics and subsequent cellular responses of drug candidates in the presence of such endogenous ligands, as the binding kinetics of these competing endogenous ligands can be substantially different^26^. For example, the binding kinetics of endogenous NK1 receptor ligands, called tachykinins, such as substance P (SP) and neurokinin A (NKA) have been found to have very divergent binding kinetics^27^. The necessity of slow receptor binding kinetics of NK1 receptor antagonists to achieve high *in vivo* efficacy in addition to the varying binding kinetics of the endogenous tachykinins, i.e. NKA and SP, makes the NK1 receptor a good model system to examine distinct kinetic interactions of antagonist and agonist binding and its effects on signal transduction.

In this study we hypothesized that the kinetic binding parameters of both the drug and the endogenous ligand play a crucial role in determining cellular responses. Hence, the *in vitro* functional effects of receptor binding kinetics were examined for kinetically divergent agonists and antagonists using the NK1 receptor as a model system. We report differential signal transduction profiles dependent on the kinetic binding characteristics of antagonists and endogenous agonists. The results were congruous throughout varying assay temperatures, cellular backgrounds, kinetic assays and a novel approach studying the onset of receptor activation. We provide, for the first time, a translation of target binding kinetics into kinetic cellular responses enabling better predictions of *in vivo* drug effects.

## Results

### Aprepitant and DFA have very divergent binding kinetics at the NK1 receptor

The kinetic binding parameters of 87 small molecule NK1 receptor antagonists were determined using a qualitative kinetic screening method, namely a dual-point competition association assay (data not shown). These experiments yielded kinetic rate index (KRI) values ranging from 0.7 ± 0.18, to 2.0 ± 0.18, i.e. indicating faster and slower dissociation kinetics in comparison to the radioligand [^3^H][Sar^9^,Met(O_2_)^11^]SP, respectively. Aprepitant (KRI of 1.8 ± 0.10) and desfluoro aprepitant (DFA) (KRI of 1.0 ± 0.13) were selected for further studies as they had the highest chemical similarity combined with the most divergent binding kinetics (Supplemental Fig. 1 and Table 1).

**Table 1:** Affinity and kinetic binding parameters of SP, NKA, DFA and aprepitant.

	pK_i_	k_on_ (nM^−1^ min^−1^)	k_off_ (min^-1^)	KRI
SP*	8.7 ± 0.01	0.24 ± 0.046	0.027 ± 0.0025	N.A.
NKA*	5.7 ± 0.04	0.0010 ± 0.00018	0.19 ± 0.036	N.A.
DFA	9.3 ± 0.04	N.A.	N.A.	1.0 ± 0.13
Aprepitant	9.5 ± 0.11	N.A.	N.A.	1.8 ± 0.10

Values are means ± SEM of at least three separate experiments performed in duplicate. *Data have been previously published in^27^.

### Real-time functional effects of NK1 receptor activation by SP and NKA are comparable between kinetic assays

**Figure 2:**
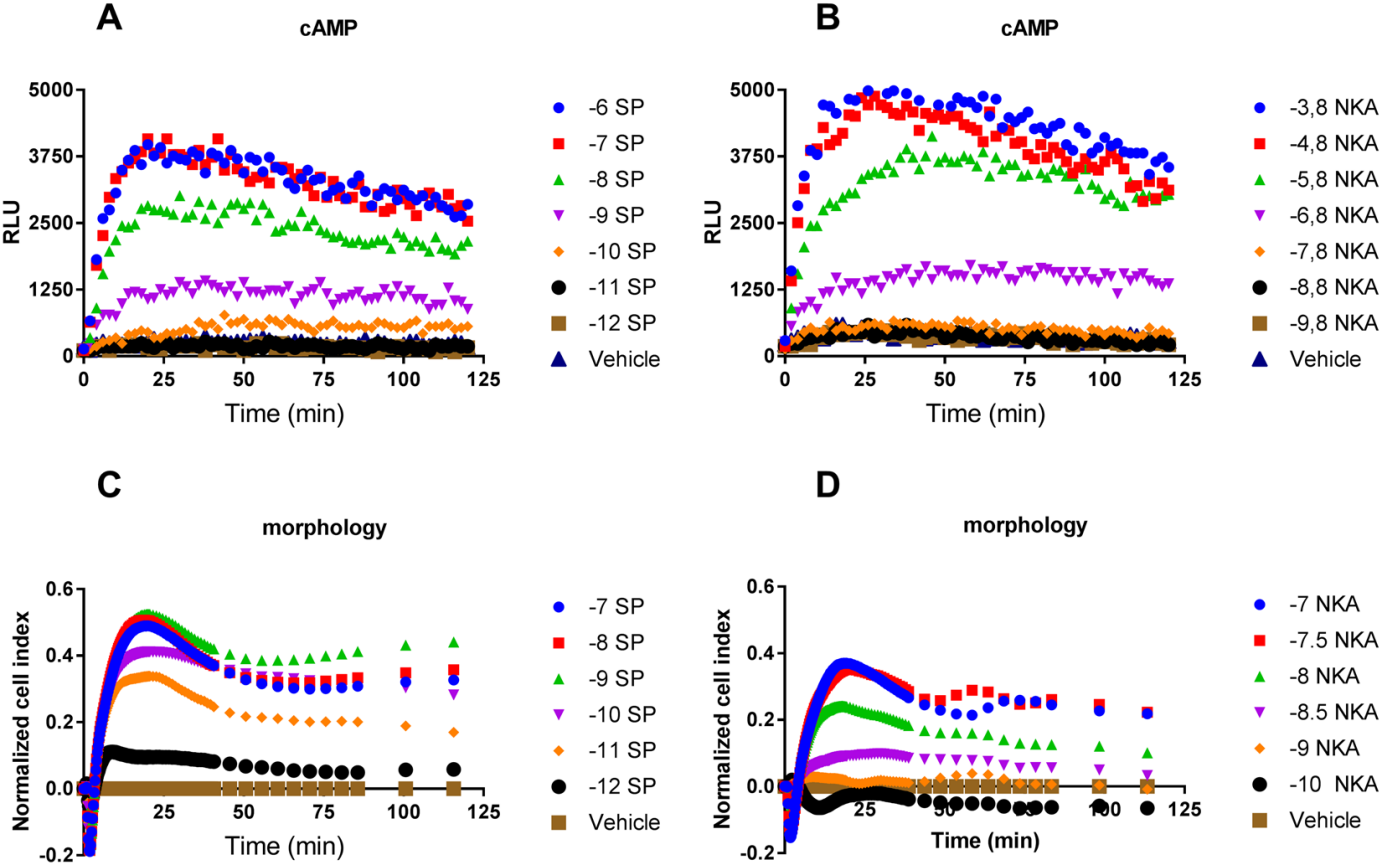
Real-time NK1 receptor-mediated responses monitored with cAMP (**A** and **B**) or morphology (**C** and **D**) experiments induced by addition of increasing concentrations of endogenous agonist SP (**A** and **C**) or NKA (**B** and **D**). Representative graph of at least three experiments performed in duplicate (morphology assays) or triplicate (cAMP assays). RLU stands for relative light units and NCI stands for normalized cell index.

The cellular response to NK1 receptor activation was monitored using two real-time assays, namely a cAMP assay (GloSensor) and a morphology-based assay (xCELLigence). A time-dependent and concentration-dependent increase in cAMP production was observed with the GloSensor assay for both endogenous agonists SP and NKA with a maximal cAMP value around 20 to 30 minutes after stimulation (Fig. 2A,B). These experiments yielded EC_50_ values for SP and NKA of 2.2 ± 0.5 nM and 483 ± 142 nM, respectively (Tables 2 and 3). Similarly, upon SP or NKA stimulation cellular impedance was increased time- and concentration-dependently with a peak response around 20–30 minutes (Fig. 2C,D), with EC_50_ values of 0.026 ± 0.004 nM for SP and 3.9 ± 1.1 nM for NKA (Table 2). Potency values obtained with the morphology assay were systematically higher in comparison to the cAMP assay.

**Table 2:** Potency and maximal effect values of SP with or without antagonist pre-incubation determined with cAMP or morphology assays.

	**EC**_**50**_ **(nM)**				**E**_**max**_ **(%)**			
	**cAMP**		**morphology**		**cAMP**		**morphology**	
	**SP**	**NKA**	**SP**	**NKA**	**SP**	**NKA**	**SP**	**NKA**
Agonist	2.2 ± 0.5	483 ± 142	0.026 ± 0.004^#^	3.9 ± 1.1^#^	100 ± 5.1	100 ± 7.7	100 ± 0.95^#^	100 ± 0.44^#^
+0.07 nM DFA	1.9 ± 0.5^NS^	399 ± 19^NS^	0.053 ± 0.020^NS^	3.0 ± 0.52^NS^	78 ± 4.5*	84 ± 18^NS^	97 ± 5.6^NS^	109 ± 7.6^NS^
+0.21 nM DFA	2.7 ± 0.8^NS^	304 ± 89^NS^	0.06 ± 0.015*	3.3 ± 0.45^NS^	75 ± 7.7*	67 ± 10*	104 ± 4.2^NS^	108 ± 6.7^NS^
+0.7 nM DFA	2.3 ± 0.7^NS^	1080 ± 356^NS^	3.0 ± 1.3*	8.5 ± 0.46*	61 ± 10**	61 ± 15*	82 ± 6.9**	81 ± 8.9**
+0.07 nM aprepitant	1.6 ± 0.2^NS^	1008 ± 165^NS^	0.022 ± 0.008^NS^	5.0 ± 1.7^NS^	62 ± 5.7**	71 ± 13^NS^	96 ± 7.6^NS^	97 ± 2.3^NS^
+0.21 nM aprepitant	8.8 + 5.4^NS^	1051 ± 170*	0.11 ± 0.01****	8.0 ± 2.8^NS^	55 ± 15**	43 ± 19**	79 ± 5.0***	79 ± 10**
+0.7 nM aprepitant	1.3 ± 0.3^NS^	4370 ± 524***	0.23 ± 0.08**	26 ± 6.8**	19 ± 5.5****	7.8 ± 4.2****	53 ± 8.5****	30 ± 6.2****

Values are means ± SEM of at least three separate experiments performed in duplicate (morphology) or triplicate (cAMP). Values were calculated with peak analysis and data were normalized to maximal response obtained for SP or NKA only. *p < 0.05, **p < 0.01, ***p < 0.001, ****p < 0.0001 compared to SP or NKA, determined using one-way ANOVA with Dunnett’s post-test. ^#^Data have been previously published in^27^.

**Table 3:** Onset of SP- or NKA-induced receptor activation after pretreatment with DFA or aprepitant determined with cAMP or morphology assays.

	**cAMP (RLU min** ^**−1**^ **)** ^**#**^		**Morphology (NCI min** ^**−1**^ **)** ^**#**^	
	**SP**	**NKA**	**SP**	**NKA**
Agonist	215 ± 37	147 ± 35	0.05 ± 0.004	0.05 ± 0.004
+0.07 nM DFA	125 ± 31^NS^	98 ± 29^NS^	0.06 ± 0.006^NS^	0.06 ± 0.005^NS^
+0.21 nM DFA	120 ± 27^NS^	98 ± 33^NS^	0.05 ± 0.006^NS^	0.05 ± 0.007^NS^
+0.7 nM DFA	158 ± 26^NS^	71 ± 22^NS^	0.03 ± 0.005*	0.04 ± 0.014^NS^
+0.07 nM aprepitant	94 ± 13*	75 ± 13^NS^	0.05 ± 0.002^NS^	0.05 ± 0.011^NS^
+0.21 nM aprepitant	63 ± 17**	43 ± 7.7*	0.04 ± 0.006^NS^	0.03 ± 0.007^NS^
+0.7 nM aprepitant	36 ± 7**	9.7 ± 1.6**	0.009 ± 0.003****	0.006 ± 0.003**

Values are means ± SEM of at least three separate experiments performed in duplicates (morphology) or triplicates (cAMP). The onset of receptor activation was calculated on the first 8 min after agonist stimulation. ^#^RLU stands for relative light units and NCI stands for normalized cell index. *p < 0.05, **p < 0.01, ***p < 0.001, ****p < 0.0001 compared to agonist only, determined using one-way ANOVA with Dunnett’s post-test.

### Aprepitant is more effective in decreasing SP-mediated maximal response

**Figure 3:**
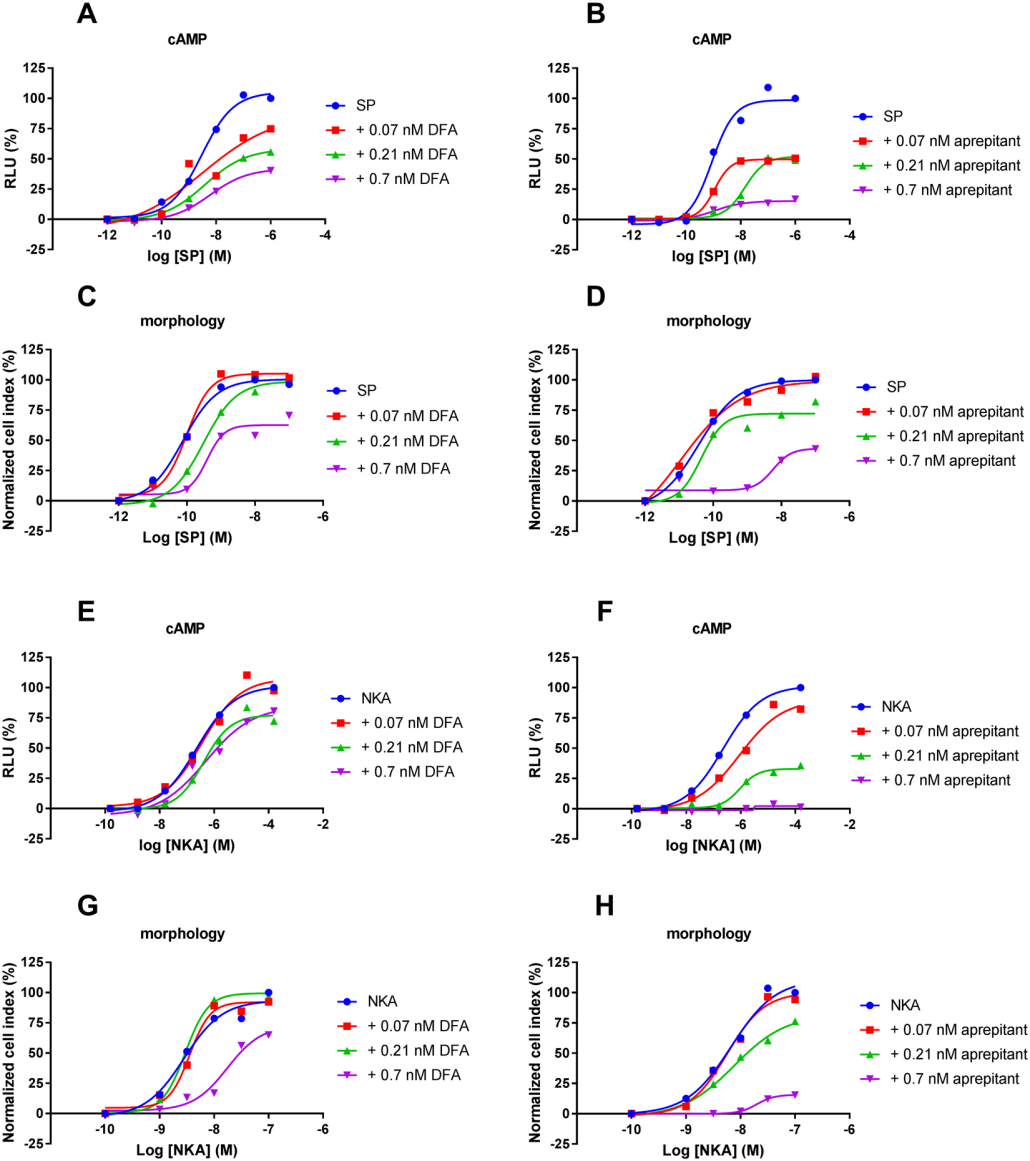
Concentration-dependent effects induced by endogenous agonist SP pre-incubated with vehicle (control), DFA or aprepitant determined with cAMP (**A** and **B**) or morphology (**C** and **D**) experiments. Concentration-dependent effects induced by endogenous agonist NKA pre-incubated with vehicle (control) DFA or aprepitant determined with cAMP (**E** and **F**) or morphology (**G** and **H**) experiments. Representative graph of at least three experiments performed in duplicate (morphology assays) or triplicate (cAMP assays). RLU stands for relative light units and NCI stands for normalized cell index.

**Figure 4:**
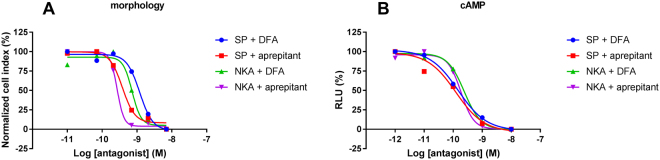
Concentration-dependent inhibition by DFA or aprepitant of EC_80_ concentrations of SP- or NKA- mediated receptor activation measured with morphology (**A**) or cAMP (**B**) experiments. Representative graph of at least three experiments performed in duplicate (morphology assays) or triplicate (cAMP assays). RLU stands for relative light units and NCI stands for normalized cell index.

To investigate the antagonistic effects of aprepitant and DFA on SP-mediated NK1 receptor activation, cells were pre-incubated with varying concentrations of antagonist prior to stimulation with SP. In the cAMP assay both antagonists were unable to significantly shift the EC_50_ of SP, however the E_max_ of SP was significantly decreased (Fig. 3A,B, Table 2). At the highest concentrations, aprepitant was more efficacious in lowering the E_max_ than DFA, abolishing it over 80% in comparison to control while DFA only decreased the E_max_ by 39% (Table 2). Interestingly, in the morphology assay both antagonists did decrease the EC_50_ of SP to 3.0 ± 1.3 nM for DFA and 0.23 ± 0.08 nM for aprepitant (Fig. 3C,D, Table 1). Similar to the cAMP assay, the E_max_ of SP was significantly reduced by the antagonists to 82 ± 6.9% in presence of DFA and to a larger extent for aprepitant to 53 ± 8.5% (Table 2). Moreover, IC_50_ values were examined by pre-incubating increasing concentrations of antagonist prior to addition of EC_80_ concentrations of agonist (Fig. 4). This resulted in IC_50_ values of 0.15 ± 0.02 nM (cAMP assay) and 0.22 ± 0.1 nM (morphology assay) for DFA. IC_50_ values for aprepitant were comparable to DFA with 0.19 ± 0.07 nM and 0.58 ± 0.22 nM from the cAMP and morphology assay, respectively.

### Antagonistic effects were more pronounced upon NKA-mediated receptor activation

The inhibitory effects of both antagonists were also investigated for NKA-mediated NK1 receptor activation. In the cAMP assay, aprepitant was able to decrease the potency of NKA by 10-fold, while DFA did not affect the agonist potency (Fig. 3E,F, Table 2). This is markedly different from the results observed with SP-mediated receptor activation. Conversely, both antagonists lowered the maximal effect of NKA, while aprepitant was most effective and lowered the E_max_ to 7.8 ± 4.2% (Table 2) and similar to SP-mediated receptor activation. With the xCELLigence, the highest concentrations of both antagonists increased the EC_50_ values by 2-fold for DFA and 7-fold for aprepitant (Fig. 3G,H, Table 2). Similar to the cAMP assay, both antagonists decreased the maximal effect of NKA while aprepitant was more efficacious (30 ± 6.2%) than DFA (81 ± 8.9%) (Table 2). Furthermore, pretreatment of increasing concentrations of antagonist prior to addition of EC_80_ concentrations of agonist resulted in IC_50_ values ranging from 0.26 ± 0.08 nM (cAMP) to 0.12 ± 0.006 nM (morphology) for DFA while IC_50_ values for aprepitant ranged from 0.23 ± 0.09 nM (cAMP) to 0.43 ± 0.07 nM (morphology) (Fig. 4). IC_50_ values of aprepitant and DFA obtained from the two assays were similar.

### Aprepitant caused a reduced rate of NK1 receptor activation induced by NKA and SP

**Figure 5:**
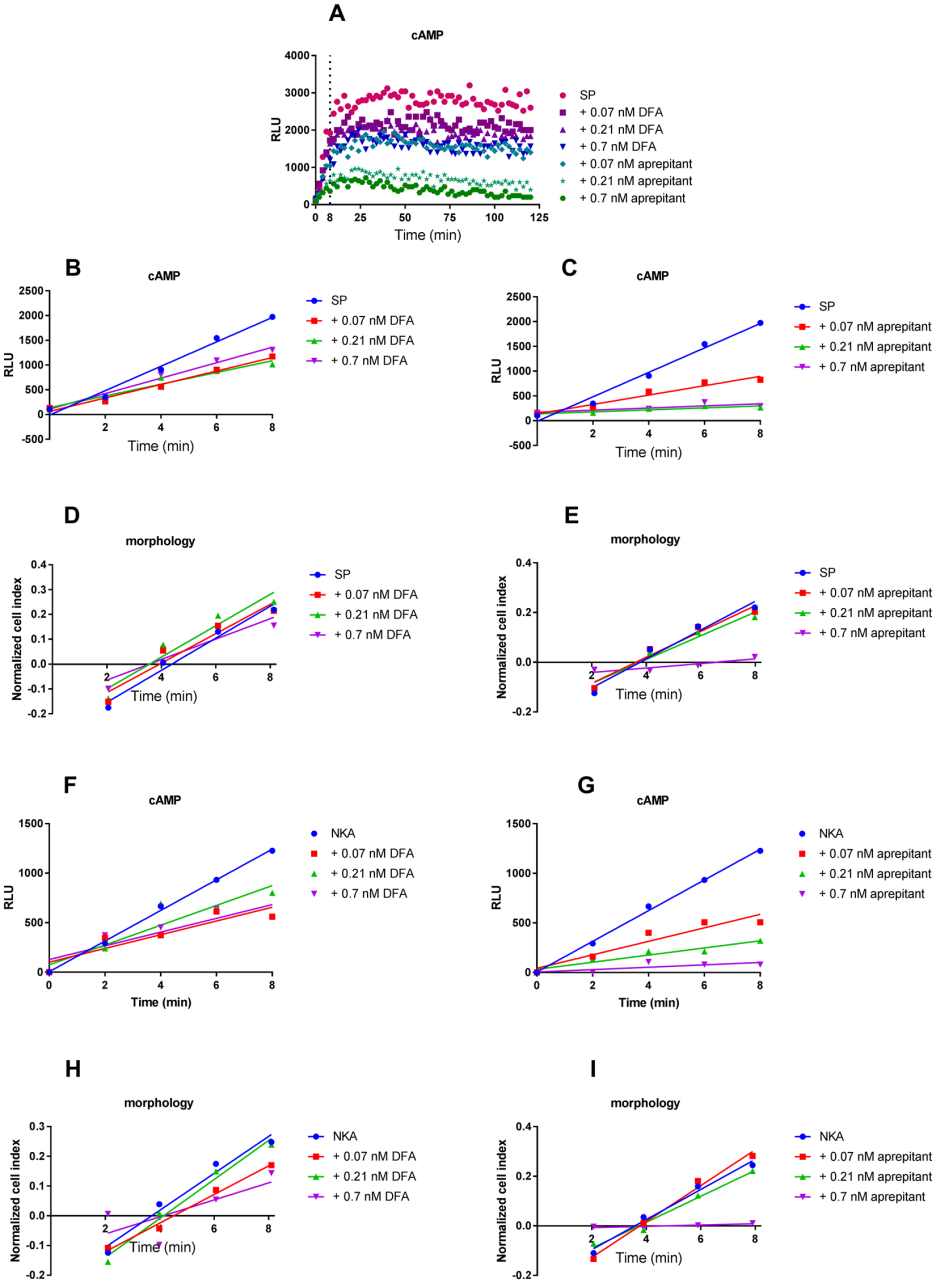
Time-dependent effects induced by EC_80_ concentration of SP after pre-incubation with DFA or aprepitant observed with cAMP assay (**A**). Zoom-in on first 8 minutes of time-dependent effects induced by EC_80_ concentration of SP (**B** and **C**) or NKA (**F** and **G**) after pre-incubation with DFA or aprepitant determined with cAMP experiments. Representative graph of at least three experiments performed in triplicate. RLU stands for relative light units. Zoom-in on first 8 minutes of time-dependent effects induced by EC_80_ concentration of SP (**D** and **E**) or NKA (**H** and **I**) after pre-incubation with DFA or aprepitant determined with morphology experiments. Representative graph of at least three experiments performed in duplicate. NCI stands for normalized cell index.

To examine the real-time effects of DFA and aprepitant on the inhibition of the cellular response to NK1 receptor activation, a novel analysis method was designed to examine the onset of receptor activation. The increase in cAMP production within the first 8 minutes after addition of the endogenous agonist was compared in the presence and absence (control) of an antagonist. The onset of SP-induced cAMP production was significantly decreased (i.e. up to 6-fold) upon pre-incubation with aprepitant but not with DFA (Fig. 5A–C, Table 3). Similarly, the onset of SP-induced impedance changes was significantly decreased 5-fold upon aprepitant pretreatment, while pretreatment with DFA was less significant (Fig. 5D,E, Table 3). Moreover, the ability of aprepitant to reduce the onset of receptor activation was more pronounced for NKA, where a significant 15-fold decrease in onset was observed in cAMP production and a significant 8-fold decrease for morphological changes (Fig. 5F–I Table 3). Conversely, DFA did not significantly decrease the onset of receptor activation in both cAMP and morphology assays.

## Discussion

To our knowledge, we are the first to provide an extensive investigation for the *in vitro* cellular responses in relation to receptor binding kinetics of antagonists and endogenous agonists. This research has significant implications for the understanding of signal transduction induced by kinetically diverse ligand-receptor interactions and the interplay between endogenous agonists and drugs targeting the receptor of interest.

The NK1 receptor is an interesting target for the treatment of neurological disorders and currently two drugs, aprepitant and netupitant, are approved for the treatment of chemotherapy induced emesis^28^. While the high *in vivo* efficacy of aprepitant is attributed to its slow dissociation kinetics^23^, a mechanistic interpretation of the translation of binding kinetics to functional effects is lacking. Therefore, this study was designed to bridge the gap between receptor binding kinetics and functional effects *in vitro*, which is important for the understanding of the translation of *in vitro* to *in vivo* data.

We hypothesized that the slowly dissociating antagonist aprepitant would be more effective in antagonizing the receptor than its fast dissociating analogue DFA. The rightward-shift in potency of SP and NKA was most discernable at the highest concentration of antagonist, where aprepitant increased the EC_50_ value and decreased the E_max_ value more significantly than DFA (Table 2, Fig. 3). Where aprepitant was fully insurmountable, DFA was only partially insurmountable. The latter can be explained by the fact that DFA is a faster dissociating compound when compared to aprepitant, but DFA is still a slower dissociating compound in comparison to the endogenous agonists SP and NKA. Hence, pre-incubation with DFA resulted in partially insurmountable antagonisms as opposed to surmountable antagonism with an even faster dissociating antagonist. Our results for aprepitant are in line with its previously reported insurmountable effects^22^. In the same study the ID_50_ values of aprepitant and DFA were determined in an animal model for CNS activity (gerbil foot tapping), where aprepitant was 3-fold more potent than its analogue DFA^22^. Another study examined the insurmountable effects of a close analogue of aprepitant and DFA, namely L-742,694. A clear decrease in E_max_ of SP after pre-incubation with L-742,694 was reported and this effect was associated with the slow dissociation rate of this antagonist from the NK1 receptor^29^. Altogether, these findings support our hypothesis that slowly dissociating antagonists are important for achieving a high *in vivo* efficacy by insurmountable antagonism at the NK1 receptor.

While both antagonists aprepitant and DFA were able to increase the EC_50_ and decrease E_max_ values for both NKA and SP, NKA was overall more sensitive to antagonism than SP. This supposed “probe-dependency”, i.e. observed effects are dependent on the probe (e.g. agonist) used, is already widely acknowledged in the field of allosteric modulation^30,31^, while this concept is rarely considered for orthosteric interactions. Interestingly, we have previously determined the binding kinetics of SP and NKA and found large differences in the association rates of both agonists, i.e. NKA associates 240-fold slower to the NK1 receptor than SP^27^. This slow association could be an explanation as to why NKA is more sensitive to antagonism, considering that both antagonists have more time to intervene with NKA target binding due to their assumed faster association rates, slower dissociation rates and pre-incubation time. The differential kinetics (and therefore sensitivity) of both endogenous agonists should be taken into account for further research towards the NK1 receptor and other GPCRs that have multiple endogenous ligands^26^.

A comparison between EC_50_ values obtained with the cAMP or morphology assays showed lower potency values for the latter. This is in line with other observations, namely that potency values acquired from label-free assays such as the xCELLigence are often reported to be much lower and may be attributed to the fact that these assays encompass the entire cellular response thereby accumulating multiple signaling pathways instead of only one^32–34^. Moreover, it appeared that the morphology assay was more sensitive to pick up shifts in potency upon antagonist treatment while the cAMP assay was most sensitive in detecting insurmountability, i.e. a decrease in maximal effect. A possible explanation could be the differences in assay set-up that can alter the assay sensitivity. For instance, morphology experiments are typically performed at 37 °C while cAMP assays were carried out at 25 °C. Lower assay temperatures result in slower dissociation rates which could explain the higher sensitivity of the cAMP assay to detect insurmountability. Moreover, the cAMP assay was carried out with CHOhNK1 cells while the morphology assay was performed with U-251 MG cells. Multiple studies have previously discussed the concept of receptor reserve, i.e. high receptor coupling efficiency and/or high-receptor density^35,36^. It was proposed that tissue with essentially no receptor reserve treated with an insurmountable antagonist could present a decrease in maximal response with only a marginal rightward shift in potency. Heterologous cell lines are often reported to have higher receptor reserves in comparison to cell lines with endogenous expressions. However, our results suggest that U-251 MG cells have a higher receptor reserve than CHOhNK1 cells and U-251 MG cells might therefore be better suited to detect a shift in potency. These findings demonstrate the importance of choosing the appropriate assay and cell type for the aim of the research.

The functional effects of antagonist binding kinetics are often examined with insurmountability assays using end-point measurements^37^ but also real-time experiments^38^. Although a few studies have paid some attention to the real-time changes in cellular effects^13,14,39,40^, we are the first to report a quantitative analysis method for the real-time cellular responses induced by agonists with antagonist pre-incubations. In this study, we were able to correlate the kinetics of receptor activation (i.e. rate of onset) to receptor binding kinetics of antagonists. The slowly dissociating antagonist aprepitant was effective in not only significantly decreasing the maximal effect of SP and NKA but also in significantly reducing the onset of receptor activation, which would have been missed using a traditional end-point assay. Hence, this novel analysis provides a robust and time-efficient screening method to detect slowly dissociating antagonists using a real-time functional assay.

In conclusion, we confirmed our hypothesis that the kinetic binding parameters of both endogenous ligand and drug play an important role in defining cellular responses. We demonstrated that the binding kinetics of both antagonists and endogenous agonists have significantly different effects on signal transduction profiles, i.e. potency values, *in vitro* efficacy values and onset rate of signal transduction. Moreover, these findings were consistent throughout different kinetic assays, assay temperatures and cellular backgrounds. We propose that incorporating real-time functional assays early in the drug discovery program will enable the detection of kinetically interesting compounds. Moreover, combining knowledge of binding kinetics and functional kinetics on drugs and endogenous ligands could improve predictions of *in vivo* drug action and thereby the success rate of drug discovery.

## Methods

### Reagents and compounds

SP and NKA were purchased from Sigma-Aldrich (St. Louis, MO) and Bio-Connect (Huissen, The Netherlands), respectively. All NK1 antagonists were synthesized in-house as described previously^22^. Chinese Hamster Ovary (CHO) cells stably expressing the human neurokinin 1 receptor (CHOhNK1 cells) were kindly provided by AstraZeneca (Macclesfield, UK) and U-251 MG cells were purchased from Sigma-Aldrich (St. Louis, MO). xCELLigence E-plate 16 and 96 were purchased from Westburg (Leusden, the Netherlands). pGloSensor™-22F cAMP plasmid, GloSensor™ cAMP reagent and FuGENE HD transfection reagent were obtained from Promega GmbH (Mannheim, Germany). CELLSTAR^®^ 384-Well Plates, Tissue Culture Treated were purchased from Greiner Bio-One (Frickenhausen, Germany). [^3^H][Sar^9^,Met(O_2_)^11^]SP (specific activity 25–55 Ci/mmol) was obtained from Perkin Elmer (Boston, MA). All other reagents and materials were obtained from commercial resources.

### Cell culture

U-251 MG cells were cultured in Earle’s Minimal Essential Medium (EMEM) supplemented with 10% FCS, 1 mM sodium pyruvate, 2 mM glutamine, 1% non-essential amino acids (NEAA), 100 IU/ml penicillin and 100 µg/ml streptomycin at 37 °C + 5% CO_2_. CHOhNK1 cells were cultured in Ham’s F12 medium supplemented with 10% fetal calf serum (FCS), 2 mM glutamine and 1 mg/ml G418 at 37 °C + 5% CO_2_.

### Dual-point competition association assays

Dual-point competition association assays were performed as prescribed previously^41^, following the radioligand binding protocol of Nederpelt *et al*.^27^. In short, CHOhNK1 membrane aliquots containing 5–15 µg protein were incubated at 4 °C with 25,000 cpm (~2.5 nM) [^3^H][Sar^9^,Met(O_2_)^11^]SP and one concentration of competing antagonist (i.e. concentration at which approximately 50% (30–70%) [^3^H][Sar^9^,Met(O_2_)^11^]SP binding was achieved). Specific binding of [^3^H][Sar^9^,Met(O_2_)^11^]SP was determined at two time-points; 30 min (t1), which is the time-point at which equilibrium of [^3^H][Sar^9^,Met(O_2_)^11^]SP binding was achieved, and 120 min (t2) at which all competing antagonists should have reached equilibrium.

### Impedance-based morphology assays

Label-free morphology assays were performed using the xCELLigence RTCA system as described previously^27,42^. U-251 MG cells were treated with three different concentrations (0.07 nM, 0.21 nM and 0.7 nM) of aprepitant or DFA for 30 min prior to stimulation with increasing concentrations of SP or NKA.

### Real-time cAMP accumulation assay

Real-time cAMP production was measured using the life cell cAMP GloSensor^TM^ assay^11,12^. The technology is based on a cAMP-biosensor, which undergoes a conformational change upon cAMP binding, followed by the turnover of Luciferin.

CHOhNK1 cells were transiently transfected with the pGloSensor™-22F cAMP (6 ng/µL) plasmid using FuGene HD (3 µL:1 µg DNA plasmid) as a transfection reagent. Accordingly, cells were harvested and reconstituted to 0.5 × 10^6^ cells/ml (10,000 cells/well) in DMEM/F-12/ HEPES supplemented with 1% FCS, 2 mM glutamine and 1 mg/ml G418. The diluted plasmid solution was combined with the transfection reagent and incubated for 20 min at room temperature. Subsequently, the transfection mixture and cell solution were mixed for additional 5 min before plating in 384-well plates. The transfected cells were incubated for 24 h at 37 °C + 5% CO_2_ followed by treatment with Glo-substrate (3% v/v) for 2 h at room temperature. Subsequently, three different concentrations (0.07 nM, 0.21 nM and 0.7 nM) of Aprepitant or DFA were added to cells for 30 min (pre-incubation) using Echo™ 550 Liquid Handler (Labcyte), followed by addition of increasing concentrations of SP or NKA. Real-time changes in the level of cAMP were detected using an Envision HTS microplate reader 2103 (PerkinElmer).

### Data analysis

All experimental data were analyzed using the curve-fitting program GraphPad Prism v. 6.00 (GraphPad Software Inc., San Diego, CA).

Data from dual-point competition association assays were analyzed by dividing the specific binding at t1 (B_t1_) with the specific binding at t2 (B_t2_).

1 $${\rm{KRI}}={{\rm{B}}}_{{\rm{t}}1}/{{\rm{B}}}_{{\rm{t}}2}$$

Data from morphology and cAMP experiments were analyzed as described previously^27^. Efficacy (E_max_) and potency (pEC_50_) values for SP and NKA were analyzed with non-linear regression of peak analysis fitted by log(agonist) vs. response - Variable slope. Results were normalized to the maximal response induced by agonist without antagonist.

The onset of receptor activation was analyzed by calculating the slope with linear regression of the first 8 minutes of the cellular response.

2 $${\rm{onset}}=\frac{{\rm{\Delta }}\,\text{cellular}\,{\rm{response}}}{{\rm{\Delta }}\,\text{time}}$$

All data are means of at least three separate experiments performed in duplicate or triplicate. Statistical analysis was performed using one-way ANOVA with Dunnett’s post-test.

## Electronic supplementary material

Supplemental Figure 1
