# Supplementary material for: From receptor binding kinetics to signal transduction; a missing link in predicting in vivo drug-action
Source: Sci Rep. 2017 Oct 26;7:14169. doi: 10.1038/s41598-017-14257-4 (PMC5658448; doi:10.1038/s41598-017-14257-4)
Supplement: Supplementary file 1 — Supplemental Figure 1 [file 41598_2017_14257_MOESM1_ESM.docx]

**From receptor binding kinetics to signal transduction; a missing link in predicting *in vivo* drug-action**

Indira Nederpelt^1^, Maria Kuzikov^2^, Wilbert E.A. de Witte^4^, Patrick Schnider^3^, Bruno Tuijt^1^, Sheraz Gul^2^, Adriaan P IJzerman^1^, Elizabeth C M de Lange^4^, Laura H Heitman^1^

^1^Division of Medicinal Chemistry, Leiden Academic Centre for Drug Research (LACDR), Leiden University, P.O. Box 9502, 2300 RA Leiden, the Netherlands

^2^Fraunhofer IME Screening Port, Schnackenburgallee 114, D-22525 Hamburg, Germany

^3^Roche Pharmaceutical Research and Early Development, Small Molecule Research, Roche Innovation Center Basel, F. Hoffmann-La Roche Ltd, Grenzacherstrasse 124, 4070 Basel, Switzerland

^4^Division of Pharmacology, Leiden Academic Centre for Drug Research (LACDR), Leiden University, P.O. Box 9502, 2300 RA Leiden, the Netherlands

**Correspondence to**: Laura H. Heitman, PhD, Gorlaeus Lab/LACDR, Leiden University, Div. Medicinal Chemistry, Einsteinweg 55, 2333 CC Leiden, The Netherlands. Tel: +31(0)71 527 4558; Fax: +31 (0) 71 527 4277; e-mail: [l.h.heitman@lacdr.leidenuniv.nl](mailto:l.h.heitman@lacdr.leidenuniv.nl)

**Supplemental Figure 1:** Chemical structure of aprepitant (A) and desfluoro aprepitant (DFA) (B).
